# A Simple Exoskeleton That Assists Plantarflexion Can Reduce the Metabolic Cost of Human Walking

**DOI:** 10.1371/journal.pone.0056137

**Published:** 2013-02-13

**Authors:** Philippe Malcolm, Wim Derave, Samuel Galle, Dirk De Clercq

**Affiliations:** Department of Movement and Sports Sciences, Ghent University, Ghent, Belgium; University of Zurich, Switzerland

## Abstract

**Background:**

Even though walking can be sustained for great distances, considerable energy is required for plantarflexion around the instant of opposite leg heel contact. Different groups attempted to reduce metabolic cost with exoskeletons but none could achieve a reduction beyond the level of walking without exoskeleton, possibly because there is no consensus on the optimal actuation timing. The main research question of our study was whether it is possible to obtain a higher reduction in metabolic cost by tuning the actuation timing.

**Methodology/Principal Findings:**

We measured metabolic cost by means of respiratory gas analysis. Test subjects walked with a simple pneumatic exoskeleton that assists plantarflexion with different actuation timings. We found that the exoskeleton can reduce metabolic cost by 0.18±0.06 W kg^−1^ or 6±2% (standard error of the mean) (p = 0.019) below the cost of walking without exoskeleton if actuation starts just before opposite leg heel contact.

**Conclusions/Significance:**

The optimum timing that we found concurs with the prediction from a mathematical model of walking. While the present exoskeleton was not ambulant, measurements of joint kinetics reveal that the required power could be recycled from knee extension deceleration work that occurs naturally during walking. This demonstrates that it is theoretically possible to build future ambulant exoskeletons that reduce metabolic cost, without power supply restrictions.

## Introduction

### Broader Context and Literature Review

At preferred speed, walking is the most metabolically economic gait mode [1] and can easily be sustained which allowed historic feats such as Roman legionnaires travelling 30 km per day [2]. This low metabolic cost is due to the total body centre of mass (COM) moving as an inverted pendulum over the stance leg, thereby allowing interchange of potential and kinetic energy [3]. Still, walking has a substantial metabolic cost as there is only up to 70% energy conservation [3].

Different groups are developing assistive robotic devices, called exoskeletons, for carrying heavy loads, assisting the mobility of the world’s aging population, *etc* [4]. Unfortunately, limited scientific information is available on the effects of these exoskeletons because of their commercial finality. In 2009, Ferris wrote a commentary article in which he expressed the need for basic studies on the physiology of exoskeleton assisted movement [5].

Several studies have been done with exoskeletons that assist plantarflexion [6]–[12]. This is a logical choice as around half of the positive muscle work during walking is delivered by the ankle [13]. However, none of these studies could produce reductions in metabolic cost below the level of normal walking without exoskeleton [6], [8], which implies that they have no practical benefit for sustained walking. Possible explanations could be that some exoskeletons are too heavy [14] and that there is no agreement on what would be the optimal actuation timing.

### Aim and Hypothesis

The overall aim of our study was to find if it is possible to reduce metabolic cost below the level of normal walking without exoskeleton by changing actuation timing during the stance phase. The only phase of walking during which high positive joint work is required is at the end of the single stance phase, when the leading leg makes heel contact [13]. The ankle extensors of the trailing leg then serve to push the COM into the next inverted pendulum arc [15]. In addition, Kuo predicted by means of a simplified mathematical model that walking is most efficiently actuated with a push off just before opposite leg heel contact [16]. To achieve our aim we conducted controlled human experiments using bilateral exoskeletons that assist plantarflexion by means of pneumatic muscles [9] (Movie S1).

### Experimental Design

A computer program permitted to trigger the onset of actuation at five increments of the stride cycle (∼13, 23, 34, 43 and 54%, (opposite leg heel contact occurs at 50%)) based on heel switch signals. Actuation offset was always at toe off (∼63%). Eight subjects walked on a treadmill wearing the exoskeleton in the five onset conditions and a reference condition with unpowered exoskeleton. We calculated metabolic power based on respiratory gas analysis [17]. In order to account for differences in assistive power due to the different conditions we also expressed the metabolic effects as a ratio versus exoskeleton power. This ratio is called exoskeleton performance index [7].

## Results and Discussion

### Metabolic Cost and Performance Index

**Figure 1:**
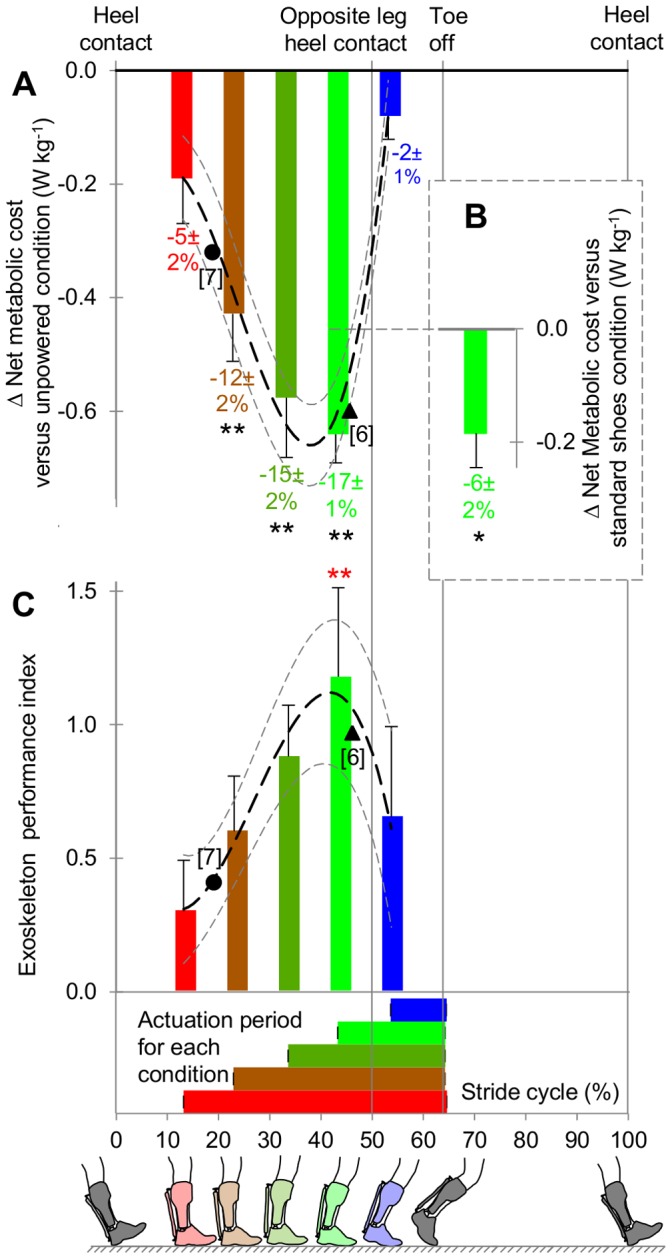
Metabolic cost and performance index. (**A**) Δ Net metabolic cost versus unpowered condition. Asterisks indicate significant differences versus unpowered condition. (**B**), 43% condition versus without exoskeleton. Asterisk indicates significant difference. (**C**) Performance index. Asterisks indicate significant difference versus 13% condition. Numbers below bars indicate differences expressed as percentages of net metabolic cost in unpowered or standard shoes condition. Horizontal bars indicate actuation duration. Vertical lines indicate heel contact and toe off. Filled circles (•) and triangles (▴) respectively indicate results derived [22], [35] from Sawicki and Ferris [7] and Norris et al. [6] (young adults population). Error bars indicate inter-subject s.e.m. Black and grey dashed lines indicate mean±s.e.m. of third-order polynomial curve fit. **p≤0.01, *p≤0.05.

We found a U-shaped pattern in metabolic cost versus actuation onset (Figure 1A). In the 43% onset condition we found the highest reduction of 0.64±0.05 W kg^−1^ (standard error of the mean (s.e.m.)) or 17±1% (s.e.m.) versus the net metabolic cost of the unpowered condition which is 3.72±0.19 W kg^−1^ (s.e.m.) (p<0.001, Tukey’s honestly significant difference (THSD) versus unpowered condition). In the same condition we found the highest performance index (p = 0.006, THSD versus 13% condition, Figure 1C). The observation that performance index is the highest in the condition with onset just before opposite leg heel contact concurs with the model of Kuo [16]. When we compared this optimal condition versus the net metabolic cost of walking without exoskeleton (3.25±0.11 W kg^−1^ (s.e.m.)) we found a reduction of 0.18±0.06 W kg^−1^ or 6±2% (s.e.m.) (p = 0.019, paired t-test, Figure 1B).

### Kinesiological Explanation

An explanation for the highest performance index in the 43% onset condition could be that the timing of the assistive power (Figure S1C) corresponds best to the biological positive ankle power as described in the literature [13], or as observed in inverse dynamical analyses with our exoskeleton (Figure S2B). It is remarkable that the optimal onset (at 43%) occurred much later than the onset of biological plantarflexor activation which occurs around 15% of the stride cycle [18]. This is probably because biological plantarflexors produce negative work by lengthening during the first part of the stance phase [18]. The exoskeleton produced almost exclusively positive work even in the earliest onset conditions by forcing the ankle angular velocity into plantarflexion (Figure S1A and C).

As such, our results suggest that a steering method based purely on biological muscle activation is not ideal for reducing metabolic cost during steady state walking with plantarflexion assisting exoskeletons with concentric actuation. The fact that Norris et al. [6] found a higher reduction in metabolic cost than Sawicki and Ferris [7] despite a much shorter habituation period (respectively 2×5 min instead of 3×30 min) could be interpreted from this perspective.

### Explanation by Step-to-Step Transition

**Figure 2:**
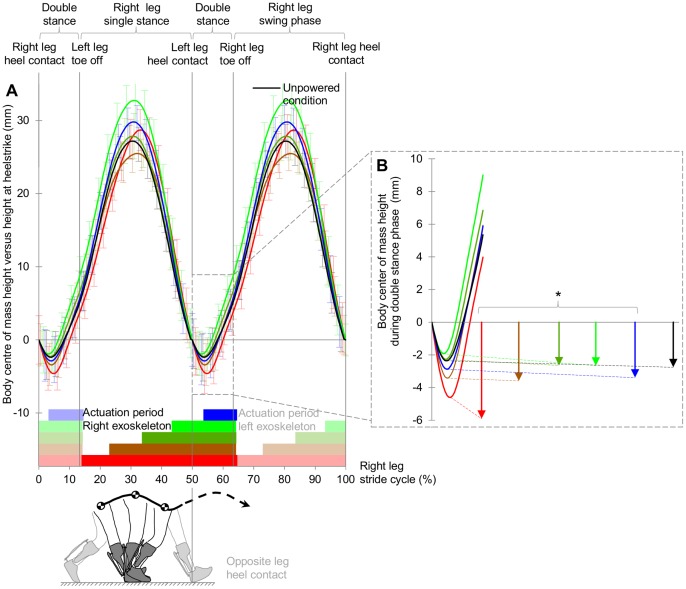
Body centre of mass (COM) height. (**A**) COM height versus height at heelstrike during right leg stride cycle. Error bars indicate inter-subject s.e.m. Opaque horizontal bars indicate actuation duration of right leg exoskeleton. Transparent bars indicate the actuation duration of opposite leg exoskeleton. Vertical lines delimit single & double stance phases. (**B**) COM height during double stance phase. Arrows indicate COM drop after heel contact. It can be noted that the arrows are slightly larger than the minima of the lines in the chart. This due to the temporal variation of the occurrence of minimum COM height is which is not shown in the lines in the chart as these only show the mean evolution of COM height. Asterisk indicates significant Pearson’s correlation between Δ COM drop versus unpowered condition and Δ net metabolic cost versus unpowered condition. Black line and arrow indicate unpowered condition. *p≤0.05.

In order to investigate the link with the inverted pendulum model we looked at the kinematics.We found that in the condition with the highest reduction in metabolic cost (*i.e.* 43% onset condition), the drop of the COM during the double stance phase (2.7±0.7 mm (s.e.m.)) was around half the size of the COM drop in 13% onset condition (5.9±0.9 mm (s.e.m.), p = 0.010, THSD versus 13% condition, Figure 2B). We also found a significant correlation of net metabolic cost with COM drop during the double stance phase (p = 0.015, R = −0.38, Pearson’s correlation of Δ’s versus unpowered condition). There was no significant correlation with vertical COM excursion during the whole stride (p = 0.832, R = 0.04). As such, the lower metabolic cost in the optimal onset condition can be attributed specifically to the COM being more effectively redirected during the step-to-step transition which experimentally validates the model of Kuo [16]. Actuation onset had no effect on the spatiotemporal parameters stride length and stride time (p>0.593, repeated measures analysis of variance, Figure S3) which could otherwise have confounded the effect of COM drop on the step-to-step transition.

### Actuation Timing Guidelines

From a practical perspective our results could be used as guidelines for steering exoskeletons. Curve fitting of the results of the tested conditions shows the highest reduction in metabolic cost could take place with onset at 37±1% (s.e.m.). This timing could be useful when power availability is not an issue, such as during rehabilitation exercise on treadmill. Also by using curve fitting, the highest performance index could be expected with onset at 45±2% (s.e.m.). This timing could be used for ambulant exoskeletons for which efficient use of power is critical. While no other study compared multiple actuation timings within the same subjects, multiple references comply with the relationship between actuation timing and metabolic parameters that we found. Results from Sawicki and Ferris [7] and Norris et al. [6] closely fit to the patterns of metabolic cost and performance index in our study (Figure 1A, C). Patients with incomplete spinal chord injuries walking with a plantarflexion assisting orthosis which they control with pushbuttons spontaneously adopt a timing that results in onset of plantarflexor torque at 43.5±3.7% (s.d.) of the stride cycle [10]. Transtibial amputees walking with a powered prosthesis subjectively indicate that opposite leg heel contact is the best time for adding power [19]. A simulation of an elastic exoskeleton shows that actuator stiffness is optimal when it allows plantarflexion to start just prior to opposite leg heel contact [20]. Altogether this suggests that an onset of concentric actuation somewhere between 40 to 50% is the most efficient for different plantarflexion assisting devices in different populations.

### Practical Significance

To illustrate the significance of the reduction that we found in net metabolic cost versus walking without exoskeleton of 6±2% (s.e.m.) this could be compared to the effect on metabolic cost of supporting 25±8% (s.e.m.) of the body weight [21]. The ability to obtain such a reduction merely by briefly acting on a distal joint could be useful to augment endurance in able bodied subjects (*e.g.* rescue workers) or to restore performance in impaired subjects (*e.g.* elderly) [4]. Based on regression formulas from the literature [22], the present reduction would allow an increase in speed of 0.05±0.02 m s^−1^ (s.e.m.), which can be categorized as a small meaningful improvement in elderly [23]. By trimming the weight of the exoskeleton (0.67 kg per side) to a realistic weight of commercial ankle-foot orthoses (0.40 kg [24]) a further reduction in metabolic cost of ∼3% [25] could be possible.

### Hypotheses on Feasibility of Ambulant Exoskeletons

The most important limitation of our exoskeleton is that it is tethered to a non-portable compressed air supply. It will probably take some time until ambulant exoskeletons will reach the same level of performance in able bodied subjects.Current studies with ambulant exoskeletons report no reductions but increases in metabolic cost going from 10 to 60% [26]–[28]. The main obstacle is power source portability [4]. It has been suggested that recycling negative work, similar to regenerative braking in hybrid cars, could be a solution for this. This solution has been proposed for the design of powered prostheses [29] and exoskeletons [12], [20], [28], [30]. While in the design of the recycling prosthesis the authors had the liberty to add moving parts wherever they wanted, in the design of a recycling exoskeleton one can only use joints that map to the able bodied human anatomy. Another limitation for exoskeletons is that only work that would otherwise be dissipated as heat should be recycled. Negative work phases followed by positive work should not be used as these are used for biological storage and return of elastic tendon energy [31]. Surprisingly, current designs for recycling exoskeletons [12], [20], [26], [28], [30] do not yet take this into account.

**Figure 3:**
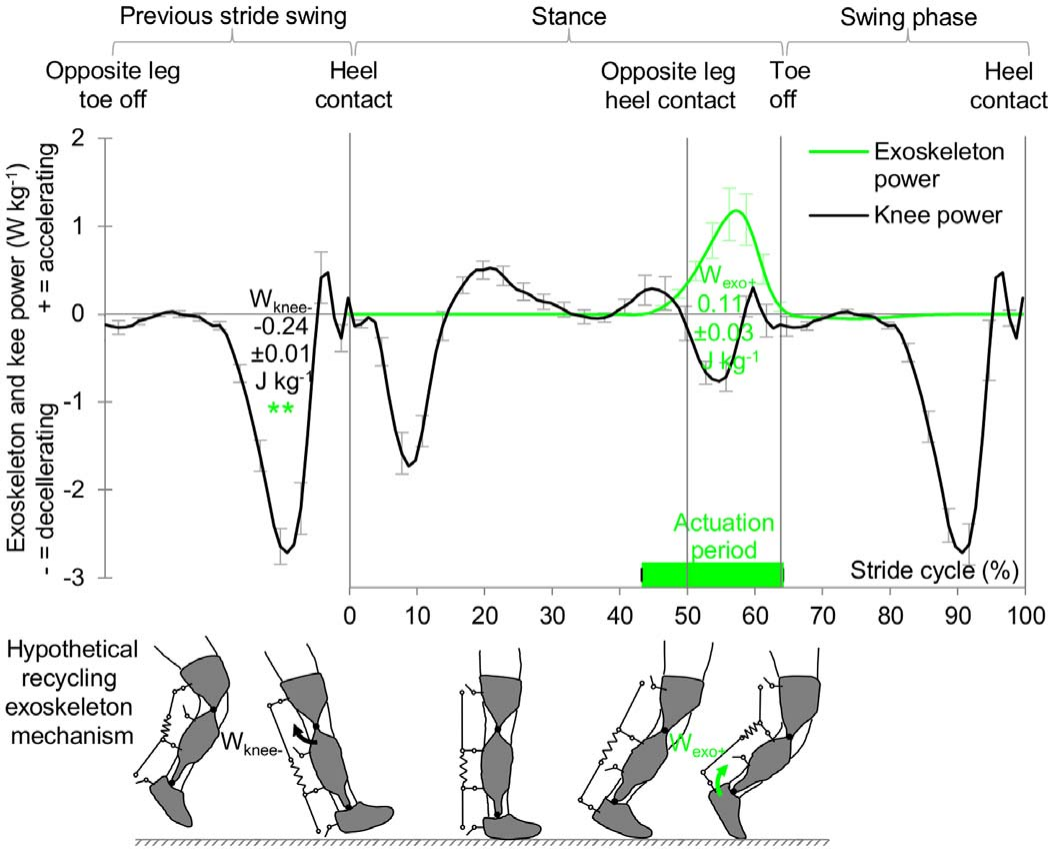
Hypothetical feasibility of an ambulant recycling exoskeleton. Black and green line respectively show knee and exoskeleton power in 43% onset condition. Numbers in chart surface indicate work during certain power peaks. Asterisks indicate significant difference between absolute value of knee swing deceleration work (W_knee−_) versus positive exoskeleton work (W_exo+_). Knee power is shown starting from the previous stride swing phase to illustrate how knee swing deceleration could be recycled into plantarflexion power during push off with a mechanism. Horizontal bar indicates actuation duration. Vertical lines indicate heel contact and toe off. Error bars indicate inter-subject s.e.m. **p≤0.01.

In order to evaluate if sufficient negative joint work would be available, we did a supplementary inverse dynamic analysis in eight subjects with the exoskeleton operating in the 43% onset condition. In the knee power we found a marked negative peak near the end of the swing phase that is not used for storage and return of tendon energy as it is followed by another negative peak [31] (Figure 3). The absolute value of the work during this phase (0.24±0.01 J kg^−1^ (s.e.m.)) was found to be significantly higher (p = 0.001, two-sample t-test) than the positive exoskeleton work in the 43% onset condition (0.11±0.03 J kg^−1^ (s.e.m.)) which means that sufficient work is available.

Future research should be oriented towards modelling of a lightweight mechanism (*e.g.* Figure 3) that can capture energy from knee swing deceleration, transfer it to the ankle and release it with optimal timing and minimal energy loss. Finally, prototype tests should be done with subjects as the human response to walking with an exoskeleton remains unpredictable [11] and the gains could be offset by the additional encumbrance. On the other hand, the substitution of negative work by the exoskeleton could provide small metabolic gains and users could also obtain additional gains by adapting their gait to optimally exploit the exoskeleton [28], [30], just like they are able to exploit biological features like biarticular muscles [18]. This training period will probably take more time than with our pneumatic exoskeleton as a recent study with an elastic exoskeleton shows continuous improvements in metabolic cost after each day of a four day training period [12].

### Conclusion

The combination of the finding that it is possible to reduce metabolic cost with a pneumatic exoskeleton and the finding that sufficient naturally occurring negative joint work is available to reproduce the power of the pneumatic exoskeleton demonstrates that it is theoretically possible to build ambulant exoskeletons that improve the metabolic economy of walking without power supply restrictions. Once this is achieved, exoskeletons could become practically useful and start to appear in everyday life as predicted by Ferris [5] and Herr [14].

## Materials and Methods

### Ethics Statement

The experiment was approved by the ethics committee of the Ghent University hospital (Belgian registration number B670220097074) and written informed consent was obtained from all subjects.

### Exoskeletons

The subjects were equipped with bilateral hinged ankle foot exoskeletons [9] (Movie S1, 0.76 kg per side) that were worn with regular shoes and powered by McKibben-type pneumatic muscles (28 cm contractile length, 3 cm diameter in relaxed state). A computer program (Labview, National Instruments, Austin, TX, USA) permitted to trigger the onset and offset of the pneumatic muscle contraction at predetermined percentages of the stride cycle based on an algorithm that predicted stride time from heel switches (IP67, Herga Electric, Suffolk, UK). The inflation pressure was 3.5 bar.

### Conditions

The onset of the actuation was set at different increments of the stride cycle (∼13, 23, 34, 43 and 54%) in five conditions. The offset of the actuation was fixed at toe off (∼63%). In the unpowered condition the subjects walked with the exoskeleton without pneumatic muscle actuation. In the standard shoes condition the subjects walked without exoskeleton but with running shoes as in Norris et al. [6]. The different onset conditions and the unpowered condition were randomized. The standard shoe condition was semi-randomized, *i.e.* it was done alternatively before or after the exoskeleton conditions.

### Subjects

We tested 10 subjects (♀, 23±1 years, 1.70±0.03 m, 66±4 kg (s.e.m.)) during treadmill walking at 1.38 m s^−1^. In a subsample of 8 subjects we recorded metabolic, kinematic, spatiotemporal and exoskeleton parameters in the five actuation onset conditions and the unpowered condition. In another subsample of 8 subjects we recorded metabolic cost during walking with standard shoes without exoskeleton and during walking with the exoskeleton operating in the 43% onset condition. In a separate sample of 8 subjects (7♀/1♂, 21±0 years, 1.67±0.02 m, 60±1 kg (s.e.m.)) we performed inverse dynamic analyses with the exoskeleton operating in the 43% onset and unpowered condition.

### Habituation

Before the measurements subjects received at least five minutes habituation to walking on treadmill and another five minutes to walking with the exoskeleton. This could be a limitation of our protocol as other studies used at least two times five minutes [6] of habituation to walking with exoskeleton. However, as mentioned in the discussion on the kinesiological explanation, exoskeletons with sufficiently sophisticated spatiotemporal control could require less habituation than exoskeletons with EMG control [32] so our subjects were probably reasonably well habituated.

### Joint Kinematics

In the treadmill experiments we recorded saggital kinematics of the right ankle joint, hip and pneumatic muscle endpoints by tracking (Maxtraq, Innovision Systems, Columbiaville, MI, USA) reflective markers from images of a 60 Hz camera (Basler AG, Ahrensburg, Germany). In the overground experiments we recorded full body 3D kinematics with a motion capture system at a rate of 200 Hz (Qualisys, Gothenburg, Sweden). We filtered the kinematics with a fourth order Butterworth lowpass filter with a 12 Hz cutoff frequency.

### Exoskeleton Kinetics

We measured the tensile force of the pneumatic muscles at a rate of 1000 Hz by means of a load cell (W2, A.L. Design, Buffalo, NY, USA) and filtered it with a fourth order Butterworth lowpass filter with a 12 Hz cutoff frequency. Actuation onset was defined as the instant when the pneumatic valve was open and pneumatic muscle force exceeded a threshold of 5 N. All the results in this paper show the measured onset so they are not affected by a potential latency of the steering system. We calculated exoskeleton power by multiplying ankle angular velocity with the pneumatic muscle moment. The pneumatic muscle moment was obtained by multiplying the tensile force with the moment arm versus the ankle.

### Metabolic Cost and Exoskeleton Performance Index

We analysed respiratory gasses from the last two minutes of each four minute treadmill condition with a computerized O_2_-CO_2_ analyser-flow meter (Oxycon Pro, Jaeger GMBH, Höchberg, Germany). We estimated metabolic cost with the formula from Brockway [17]. We calculated net metabolic cost by subtracting the metabolic cost measured during the last two minutes of four minutes standing still before the experiments from the metabolic cost in the experimental conditions. In order to specifically reflect the effects of the assistance from the exoskeleton, results were reported as the difference versus the unpowered condition and versus the standard shoes condition. In order to reflect the efficiency of the exoskeleton we calculated an index proposed by Sawicki and Ferris [7].
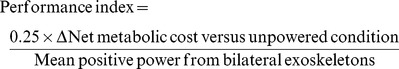
(1)


### Body Centre of Mass Height

We estimated the change in COM height based on the change in vertical position of pelvis [33]. The change in vertical position of the pelvis was calculated by taking the mean of the vertical position of the right hip and the estimated vertical position of the left hip obtained from shifting the right hip vertical position by half a stride.

### Joint Kinetics

In order to be able to measure ground reaction forces we let subjects walk over at walkway with an embedded forceplate (Kistler, Winterthur, Switzerland). A similar walking speed as in the treadmill experiments was imposed by means of pacing lights alongside the walkway. The compressed air supply and the exoskeleton steering unit were rolled in a cart behind the subject. Sagittal ankle and knee joint kinetics were calculated with dedicated software (Visual3D, C-motion, Rockville, MD, USA) based on ground reaction forces, 3D kinematics and a segmental mass distribution model from Dempster [34] that was modified to incorporate the parts of the exoskeleton.

### Spatiotemporal Parameters

In the treadmill experiment we determined initial contact and toe off from images of the 60 Hz camera. Step length was derived based on the treadmill speed. In the overground experiments we determined initial contact times based on the ground reaction forces and kinematics.

### Statistics

We opted for parametric statistics in order to obtain comparable results as other studies on metabolic cost of walking with plantarflexion assisting exoskeletons with similar sample sizes (*i.e.* 9 subjects) [6]–[8]. In the first subsample of subjects we analysed the overall effect of actuation timing on net metabolic cost, performance index and COM drop with a repeated measures analysis of variance. In order to estimate at what onset timing the highest reduction in metabolic cost and the highest performance index would be situated between the tested conditions we calculated the maxima of third order polynomial curve fits versus actuation timing (R^2^ = 0. 91±0.06 (s.e.m.) for metabolic cost and R^2^ = 0.80±0.07 (s.e.m.) for performance index, coefficients of determination). Differences between conditions were analysed with a Tukey’s honestly significant difference test. Correlations of metabolic cost versus COM kinematic parameters were analysed with Pearson’s correlation. In the second subsample of subjects we analysed the difference in metabolic cost of the 43% onset condition versus the standard shoes condition with a paired t-test. In the sample of subjects who were tested overground we compared absolute value of the knee swing deceleration negative work versus the exoskeleton positive work from the optimal 43% onset condition from the treadmill experiments with a two-sample t-test. For all tests we used 8 subjects, two-tailed p-values and an alpha level of 0.05.

## Supporting Information

Figure S1 **Kinematics and exoskeleton kinetics.** (**A**) Ankle joint angular velocity. Black line indicates unpowered condition. (**B**) Exoskeleton moment. (**C**) Exoskeleton power. Error bars indicate inter-subject s.e.m. Horizontal bars indicate actuation duration of exoskeleton. Vertical lines indicate heel contact and toe off.
(TIF)

Figure S2 **Ankle joint kinetics in 43% condition (green lines) versus unpowered condition (black lines).** (**A**) Total ankle joint moment. (**B**) Total ankle joint power. Error bars indicate inter-subject s.e.m. Horizontal bar indicates actuation duration of exoskeleton. Vertical lines indicate heel contact and toe off.
(TIF)

Figure S3 **Spatiotemporal parameters**. Left axis shows stride length and right axis shows stride time. Black bar shows unpowered condition. Error bars indicate inter-subject s.e.m. Horizontal bars indicate actuation duration of exoskeleton. Vertical lines indicate heel contact and toe off.
(TIF)

Movie S1 **Exoskeleton operating in 43% onset condition.** Saggital video of a representative subject (half of actual speed). It can be observed how the exoskeletons assist plantarflexion by means of the contraction of the pneumatic muscles mounted on the rear.
(MP4)
