# Supplementary figures and images for: A Simple Exoskeleton That Assists Plantarflexion Can Reduce the Metabolic Cost of Human Walking
Source: PLoS One. 2013 Feb 13;8(2):e56137. doi: 10.1371/journal.pone.0056137 (PMC3571952; doi:10.1371/journal.pone.0056137)

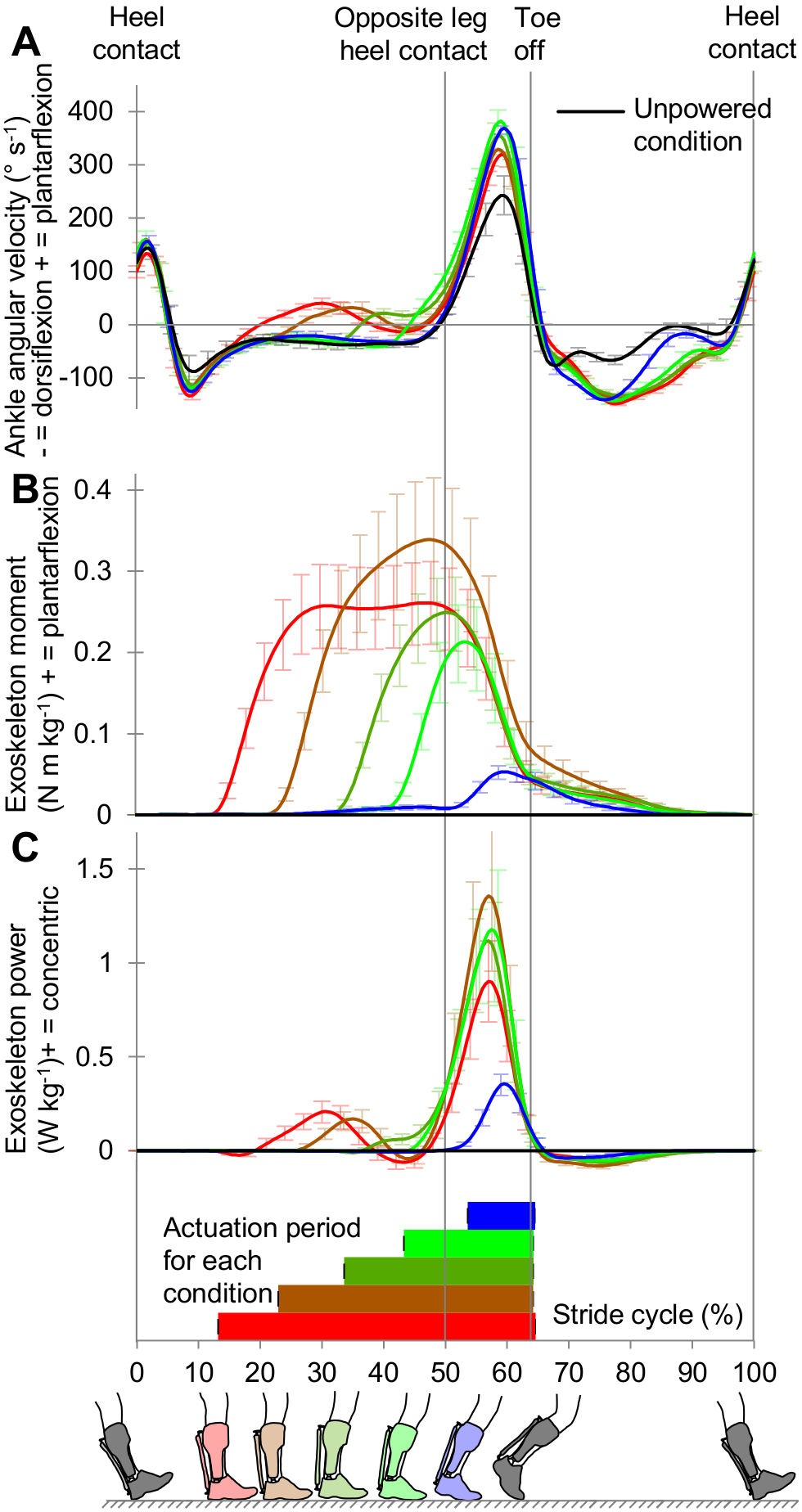

Supplement: Figure S1 — Kinematics and exoskeleton kinetics. (A) Ankle joint angular velocity. Black line indicates unpowered condition. (B) Exoskeleton moment. (C) Exoskeleton power. Error bars indicate inter-subject s.e.m. Horizontal bars indicate actuation duration of exoskeleton. Vertical lines indicate heel contact and toe off. (TIF) [file pone.0056137.s001.tif]

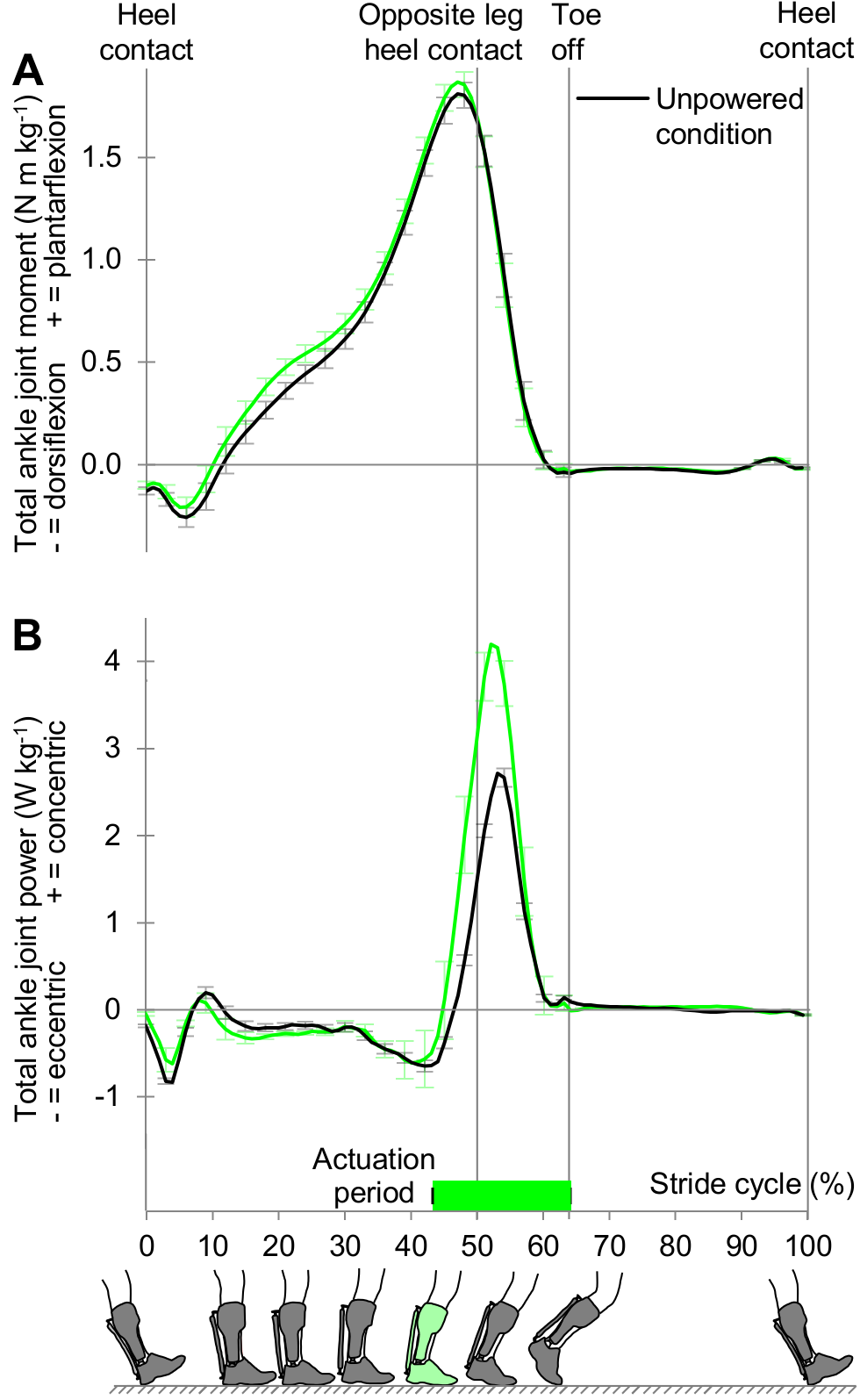

Supplement: Figure S2 — Ankle joint kinetics in 43% condition (green lines) versus unpowered condition (black lines). (A) Total ankle joint moment. (B) Total ankle joint power. Error bars indicate inter-subject s.e.m. Horizontal bar indicates actuation duration of exoskeleton. Vertical lines indicate heel contact and toe off. (TIF) [file pone.0056137.s002.tif]

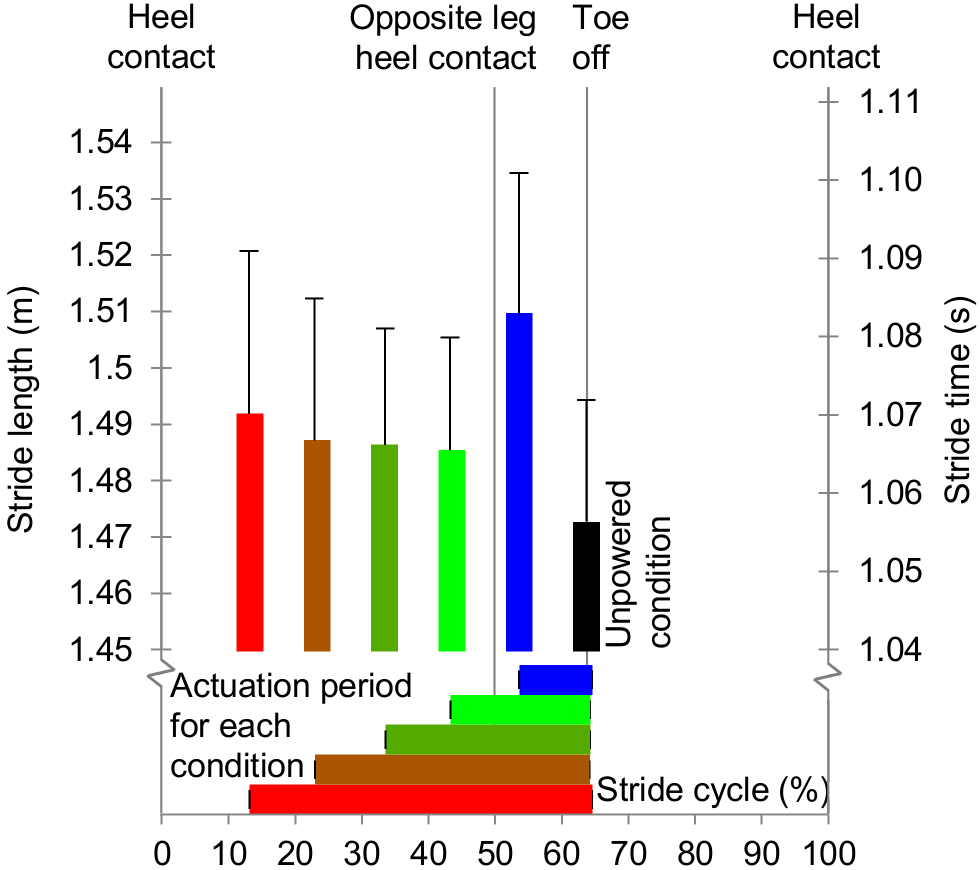

Supplement: Figure S3 — Spatiotemporal parameters. Left axis shows stride length and right axis shows stride time. Black bar shows unpowered condition. Error bars indicate inter-subject s.e.m. Horizontal bars indicate actuation duration of exoskeleton. Vertical lines indicate heel contact and toe off. (TIF) [file pone.0056137.s003.tif]
